# Molecular Characterization of Knock‐In Mouse Models Harboring Genetic Risk Factors for Late‐Onset Alzheimer’s Disease

**DOI:** 10.1002/alz.086533

**Published:** 2025-01-03

**Authors:** Gregory W Carter

**Affiliations:** ^1^ The Jackson Laboratory, Bar Harbor, ME USA

## Abstract

**Background:**

Determining the precise genetic mechanisms that contribute to LOAD, both in coding and noncoding variants, will enable a deeper understanding of pathogenesis and advance preclinical models for the testing of targeted therapeutics.

**Methods:**

We have introduced candidate genetic variants in the *EPHA1*, *BIN1*, *CD2AP*, *SCIMP, KLOTHO, PTK2B, ADAMTS4, IL1RAP, IL34*, and *PTPRB* loci into a sensitized mouse model already harboring humanized amyloid‐beta, APOE4, and Trem2.R47H alleles knocked in to a C57BL/6J background. Variants were selected based on predicted function, cross‐species conservation, increased risk of LOAD, and allele frequency. Coding variants were chosen in *PTPRB, IL34*, and *KLOTHO* while promoter and/or enhancer variants were modeled for the remaining loci. Genome editing with CRISPR‐Cas9 was performed and mouse cohorts were aged to four and 12 months. Homogenized brain hemispheres were assayed from both male and female mice with RNA‐seq. Transcriptomic changes were compared to postmortem human brain data to determine disease relevance.

**Results:**

Transcriptomic effects from these genetic variants recapitulated a variety of human gene expression patterns observed in LOAD study cohorts, particularly when signatures were partitioned into Alzheimer’s relevant biodomains. By 12 months of age, PTPRB*D57N mice exhibited neuroimmune signatures that correlate with postmortem LOAD cases relative to controls. A noncoding variant in the *EPHA1* region primarily altered apoptotic processes, potentially driven by expression changes in *CASP2* at the locus. The *BIN1* promoter variant exhibited both neuroimmune and oligodendrocyte‐related changes that correlated with LOAD modules. These changes were more pronounced with age, supporting their role in age‐related dementia.

**Conclusions:**

We have characterized in vivo signatures of nine genetic candidates for LOAD, identifying alterations in specific LOAD‐related pathways in each variant on a sensitized genetic background. These results provide animal models for preclinical testing of therapeutics designed to correct specific molecular alterations that contribute to LOAD pathology and progression.

